# The effect of lateral wall perforation on screw pull-out strength: a cadaveric study

**DOI:** 10.1186/s13018-015-0157-0

**Published:** 2015-01-24

**Authors:** Nan Li, Da He, Yonggang Xing, Yanwei LV, Wei Tian

**Affiliations:** Department of Spine Surgery, Beijing Jishuitan Hospital, 31 Xinjiekou Dongjie, Beijing, 100035 China; Clinical Statistics and Epidemiology Research Office, Traumatology and Orthopaedics Research Institute of Beijing, Beijing Jishuitan Hospital, Beijing, China

**Keywords:** Lumbar spine, Pedicular screw insertion, Pull-out strength

## Abstract

**Background:**

Lateral pedicle wall perforations occur frequently during pedicle screw insertion. Although it is known that such an occurrence decreases the screw pull-out strength, the effect has not been quantified biomechanically.

**Materials and methods:**

Twenty fresh cadaveric lumbar vertebrae were harvested, and the bone mineral density (BMD) of each was evaluated with dual-energy radiography absorptiometry (DEXA). Twenty matched, 6.5-mm pedicle screws were inserted in two different manners in two groups, the control group and the experimental group. In the control group, the pedicle screw was inserted in a standard fashion taking adequate precaution to ensure there was no perforation of the wall. In the experimental group, the pedicle screw was inserted such that its trajectory perforated the lateral wall. Group assignments were done randomly, and the maximal fixation strength was recorded for each screw pull-out test with a material-testing system (MTS 858 II).

**Results:**

The average BMD for both groups was 0.850 g/cm^2^ (0.788–0.912 g/cm^2^). The average (and standard deviation) maximal pull-out forces were 1,015.8 ± 249.40 N for the experimental group and 1,326.0 ± 320.50 N for the control group. According to a paired *t*-test, the difference between the two groups was statistically significant (*P* < 0.001).

**Conclusion:**

The results of this study confirm that the maximal pull-out strength of pedicle screws decreases by approximately 23.4% when the lateral wall is perforated.

## Introduction

The use of pedicle screws in the lumbar region is a well-established technique that has been shown to provide immediate stability and rigid fixation that facilitates correction of a deformity in both sagittal and coronal planes [1-5]. However, to ensure optimal placement for achieving the requisite stability, the screw must be meticulously placed and insertion obtained in good quality bone. Various techniques have been developed to ensure optimal placement of pedicle screws in the pedicles. In the straight-ahead technique as described by Roy-Camille [6], screw insertion begins at the intersection of a horizontal line bisecting the transverse process and a longitudinal line bisecting the facet joint. The screw is then inserted straight ahead, parallel to the vertebral endplates. The Magerl [7] technique uses the same horizontal landmark for screw insertion as the Roy-Camille technique, but for the longitudinal line, the landmark is just lateral to the angle of the superior facet. The screw is then angled laterally to medially while kept parallel to the vertebral endplates. The up-and-in method of screw placement uses the same longitudinal reference line as described by Magerl et al., but with a horizontal reference line that crosses the lower third of the transverse process. The screws are then placed in a caudad-to-cephalad direction toward, but not into, the vertebral endplate. The screws are also angled slightly medially as in the Magerl technique [8]. Beyond the conventional techniques using intraoperative landmarks, recent advancements in a developed navigation technique have begun to help surgeons insert pedicle screws more accurately [9-14]. However, despite experience with conventional techniques and advancements in the field of intraoperative navigation, intraoperative lateral pedicular wall perforation is not uncommon. This has been attributed to the morphology of the pedicle and also the fact that the lateral wall is the weakest among all walls making up the pedicle [15]. Castro [16] reported that 14 (11%) of 131 screws penetrated the lateral wall of the pedicle in 30 patients after lumbar spinal fusion, as assessed using computed tomography. Silbermann [9] compared the accuracy rates of pedicle screw placement between the free-hand and O-arm-based navigation techniques and found that 34 (22.4%) of 152 screws showed medial encroachment and 14 (9.2%) screws showed lateral encroachment with free-hand placement in comparison to 2 (1.1%) and 7 (3.7%) of 187 screws, respectively, with O-arm-based navigation. Thus, lateral perforation seemed more likely to occur with O-arm-based navigation than with free-hand placement. Galalis [17] also published a systematic review comparing free-hand placement, fluoroscopy guidance, and navigation techniques. Twenty-six prospective clinical studies were included in the analysis, and these studies included 1,105 patients in whom 6,617 screws were inserted. When evaluating the position of perforation, in the studies using the free-hand technique, a range from 12 to 67% was found for lateral perforation. When fluoroscopy was used, the pedicles were perforated laterally with an incidence of 16 to 79%. In patients in whom computed tomography (CT) navigation was used, the proportion of screws that perforated the lateral wall was significantly increased, ranging from 29 to 80%, compared to the percentage of screws that perforated the medial wall, which ranged from 8 to 29%. All of these studies suggest that meticulous attention should be paid to the lateral placement of pedicle screws, especially when using navigation and assistance techniques, as with these the incidence of lateral perforation is significantly higher. In addition to biomechanical changes, lateral wall perforation may also result in vascular injuries, especially when the aorta and other retroperitoneal structures are located close to the screw trajectory and the vertebral bodies [18,19]. Anatomic studies have shown that, even in severe scoliosis, the aorta persistently follows and adheres to the abnormal curves of the spine [20]. Many studies on the accuracy of the screw trajectory and its actual placement in the pedicle have revealed that a large percentage of screws penetrate the pedicle lateral cortex, placing major vascular structures at risk for injury [21]. Many case series and reports have reported similar findings, and Minor [22] reported a case of a patient who underwent surgical correction of a spinal deformity and had to receive endovascular treatment following iatrogenic injury. Postoperative CT scans of the case revealed a laterally misplaced pedicle screw, which was impinging on the descending aortic wall. The patient was brought to the operating room, where a thoracic stent graft was deployed under fluoroscopic guidance as the malpositioned screw was manually retracted. Although vascular injuries associated with spinal surgery have delayed presentation, occurring only after chronic irritation of the pulsating aortic wall against a metallic implant, immediate intervention is still indicated to prevent potentially serious, future complications.

It is thought that lateral pedicle wall perforation negatively impacts the purchase of the screw in the pedicle and may consequently reduce its pull-out strength. However, there is still a lack of quantitative evidence demonstrating this negative correlation. Thus, the present biomechanical study was undertaken to explore and quantify the impact of perforation of the lateral wall during pedicle screw insertion on its pull out strength.

## Material and methods

Requisite institutional review board (IRB; Beijing Jishuitan Hospital ethnics committee) approvals were obtained for the study. For the purpose of the study, 20 freshly frozen lumbar vertebrae (L1-L5) were harvested from 4 cadavers (3 males, 1 female) with an average age of 54.0 years (range, 47–67 years). Each vertebra was dissected individually and then carefully inspected to ensure that all the specimens were free from metastatic or any obvious metabolic bone disease. Furthermore, the vertebrae were visually examined to eliminate vertebrae with previous fracture or instrumentation. Dual-energy radiography absorptiometry (DEXA) was then performed to measure bone mineral density (BMD) using a Lunar Prodigy, encore 2006 instrument (General Electric, Madison, WI, USA). As all the specimens were from middle-aged adults, osteophyte formation and facet hypertrophy were minor. Thus, DEXA scanning used for BMD measurement offers greater precision in this study than in other studies in which the specimens were obtained from relatively older individuals.

**Figure 1:**
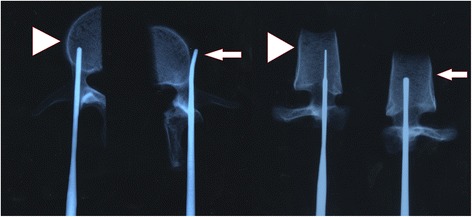
**AP and lateral X-ray of the experimental (long arrow) and control (arrow head) group.** The probe traveled out of the pedicle through the breakage in the lateral wall in the experimental group.

**Figure 2:**
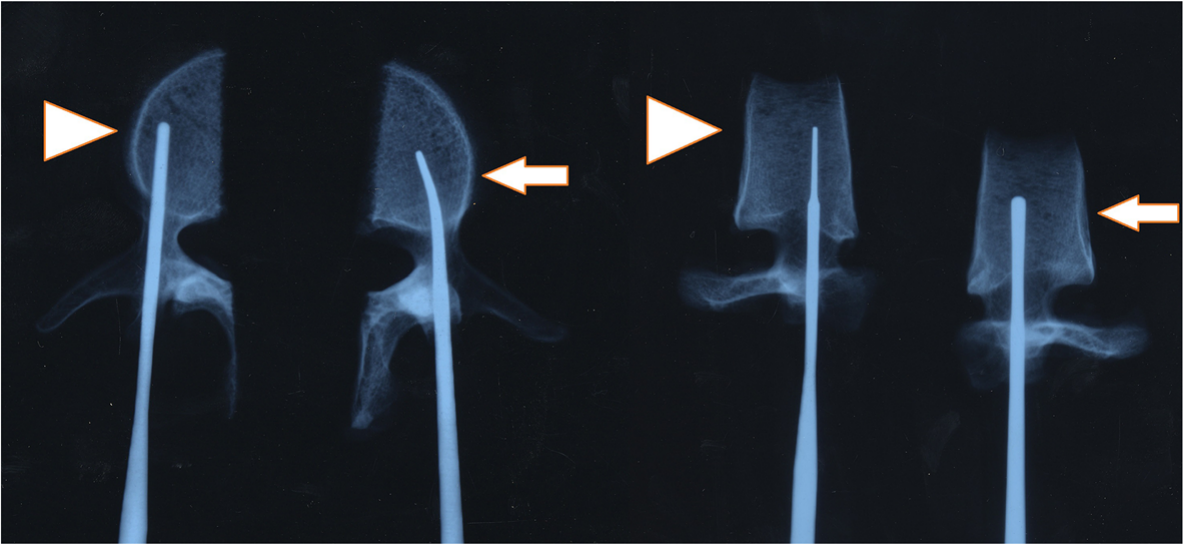
**AP and lateral X-ray images show that the probe re-entered the vertebral body after changing direction.** Control group, arrow head; experimental group, long arrow.

**Figure 3:**
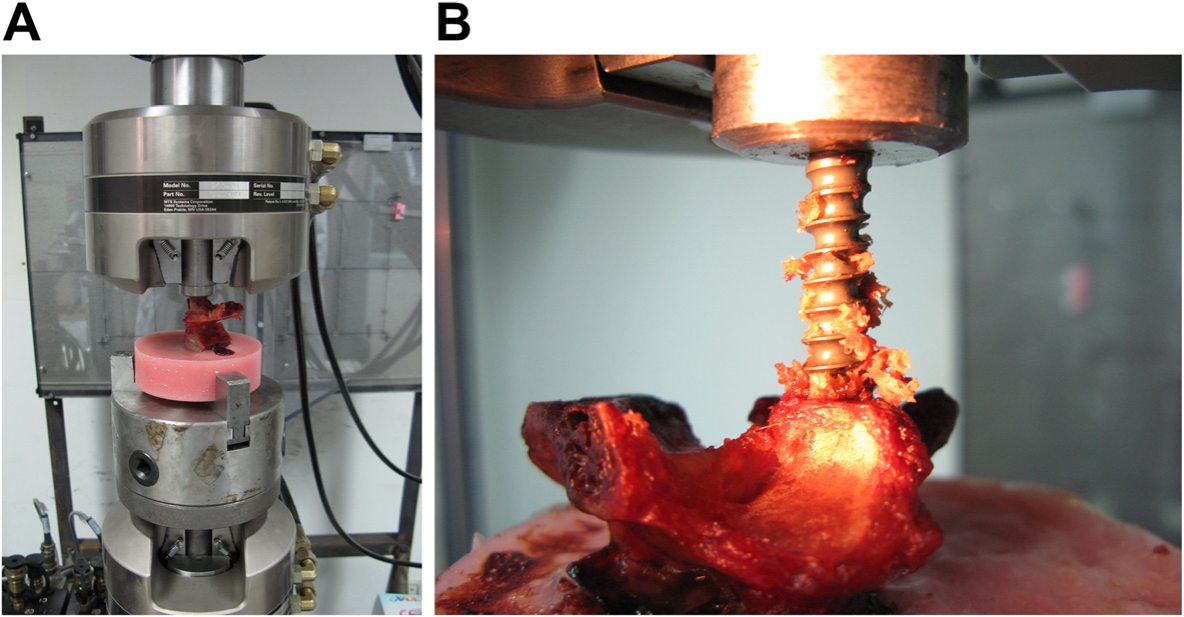
**Mono-axial pedicle screw and pedicle screw pull-out strength. A** The mono-axial pedicle screw (6.5 mm × 45 mm, M8, Sofamor-Danek, USA) was used to fix the vertebral body and connected to a multi-functional biomechanical testing machine (MTS 858). **B** The pedicle screw pull-out strength was tested using the MTS at a rate of 5 mm/min.

Before testing, the specimens were thawed for 24 h to room temperature from their storage temperature of −30°C and cleaned of all soft tissues. Each vertebra was then cut into two halves along the sagittal midline using an electric saw. Individual pedicles were randomly assigned to two groups: the control group and the experimental group. For the control group, the standard procedure (Magerl method) for pedicle screw insertion was used. A 3.5-mm burr was used to create a pilot hole on the dorsal cortex for screw entrance. The entire pedicle tract was then probed with the universal device into the vertebral body to a depth of about 40 mm, and an X-ray/image intensifier was used during the procedure to ensure the proper trajectory and avoid cortical perforation. In contrast, on the contralateral side that formed the experimental group, the lateral cortex at the junction between the pedicle and vertebral body was perforated with a 3.0-mm burr before the probe passed out of the pedicle through this hole, in order to mimic the lateral perforation that occurs incidentally during the live surgery. The pedicle was then probed as in the control group after changing the transverse angle. Each vertebra was then instrumented with 6.5 × 45-mm M8 titanium alloy screws (Medtronic, Sofamor-Danek, Memphis, TN) (Figures 1 and 2). Subsequently, for the purpose of biomechanical analysis, all vertebrae were embedded in bone cement after confirming that each screw was fully contained inside the pedicle. In order to ensure that bone cement did not accidentally enter the lateral perforation in the experimental group, these perforations were sealed with plasticine. Finally, the pull-out strength of each pedicle screw in both groups was measured with a MTS-858 II material tester (Material Testing System Corporation, Minneapolis, MN), which was connected to the screw through a jig to align the pull out direction along the longitudinal axis of the screw. This ensured that any load from the other direction was eliminated (Figure 3A, B). The screw was pulled out at a constant velocity of 5 mm/min, and the peak load was taken as the pull-out strength.

BMD and pull-out strength were compared between the two groups using paired *t*-tests. *P* < 0.05 indicated a statistically significant difference. All data were analyzed using SPSS 14.0 software (SPSS Inc., Chicago, IL).

## Results

**Figure 4:**
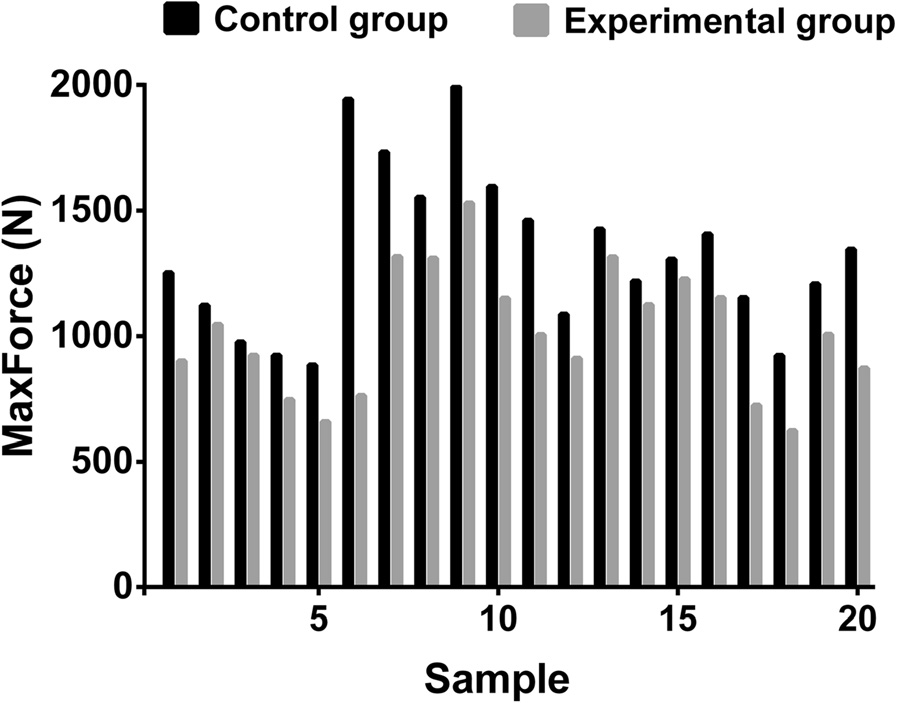
**The maximum pull-out strength in both the experimental and control group.**

The average BMD of the 20 specimens was 0.850 ± 0.062 g/cm^2^. Differences in BMD between individual vertebrae were not statistically significant, which showed the specimens were normal and not affected by osteoporosis. In both the experimental and control groups formed from 20 vertebral bodies (from 4 cadavers), the fixation strength was measured (Tables 1 and 2). The results of paired *t*-tests showed that the pull-out strength was significantly greater in the control group than in the experimental group (1,326.0 ± 320.50 vs 1,015.8 ± 249.40 N, *P* < 0.001; Figure 4). The average value in the experimental group was about 76.6% of that in the control group.

**Table 1:** **The fixation strength of the pedicle screw to the different vertebral bodies in no. 1 and 2 cadavers**

**VB**	**Cad 1 (B) (N)**	**(A) (N)**	**Cad 2 (B) (N)**	**(A) (N)**
L1	901	1,253	1,316	1,733
L2	1,047	1,124	763	1,945
L3	924	977	1,152	1,596
L4	747	924	1,229	1,305
L5	659	885	911	1,088

B, experimental group; A, control group.

**Table 2:** **The fixation strength of the pedicle screw at different vertebral bodies in the no. 3 and 4 cadavers**

**VB**	**Cad 3 (B) (N)**	**(A) (N)**	**Cad 4 (B) (N)**	**(A) (N)**
L1	1,309	1,553	1,006	1,461
L2	1,530	1,993	1,315	1,426
L3	1,154	1,406	1,007	1,209
L4	1,125	1,220	873	1,345
L5	623	923	724	1,154

B, experimental group; A, control group.

**Figure 5:**
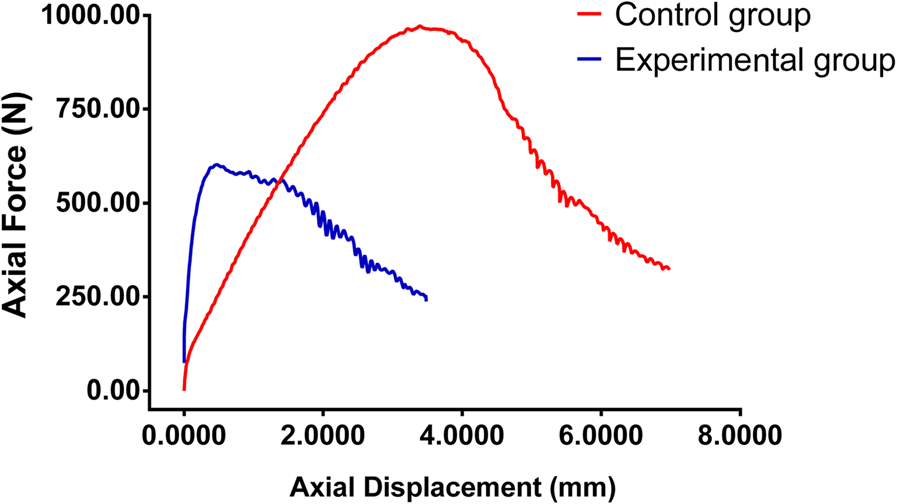
**Fixation strength in both experimental and control group from L5 vertebral body in cadaver vertebra no. 3.**

**Figure 6:**
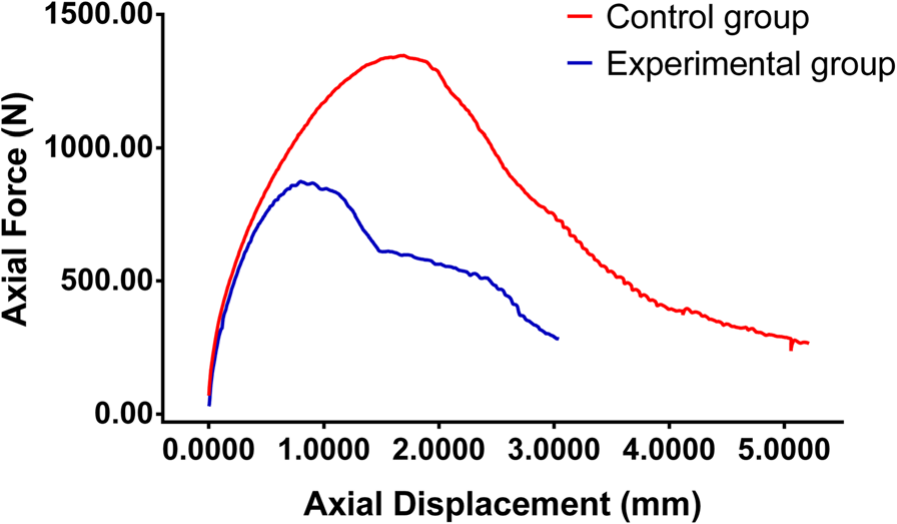
**Fixation strength in both experimental and control group from L4 vertebral body in cadaver vertebra no 4.**

For more detailed analysis, two vertebral bodies at different levels were selected randomly and compared separately. One was the L5 vertebra from cadaver no. 3, for which the pull-out strength was 623 N in the experimental group and 923 N in the control group. The other was the L4 vertebra from cadaver no. 4, for which the pull-out strength was 873 N in the experimental group and 1,345 N in the control group. The data from the two vertebrae were plotted with the axial pull-out strength along the *y*-axis and axial displacement along the *x*-axis (Figures 5 and 6).

## Discussion

Correct placement of transpedicular screws for spinal fusion is technically challenging due to several factors such as the variable anatomy of vertebral bodies, the relatively narrow pedicle in some thin patients at Asia, and the complicated three-dimensional orientation of the pedicle, especially in cases of scoliosis. Therefore, pedicle perforation and screw misplacement occurs frequently in clinical practice [23-25]. Perforation may weaken the fixation strength of screws in vertebrae, particularly in cases with lateral cortical perforation. George et al. [26] observed that unintentional pedicle fracture reduces the mean pull-out strength by 11% compared to that of screws in intact pedicles. Saraf et al. [27] observed that the mean pull-out strength of laterally misplaced screws was 47.3% less than that of standard pedicle screws in the thoracic and lumbar vertebrae through a cadaveric study, but did not find any correlation between BMD and ultimate pull-out strength. Brasiliense et al. [28] also reported that laterally misplaced pedicle screws have a 21% lower pull-out strength compared to well-placed pedicle screws, although their study included only thoracic human cadaveric vertebrae.

Frequent disruption of the lateral pedicle wall can be attributed to the anatomy of the pedicle, which is a cylindrical body located between the vertebral body and lamina. It is also due to the fact that the lateral wall is the weakest of all the walls of the pedicle. Weinstein et al. [29] demonstrated that during screw fixation of thoracic or lumbar vertebral body, the pedicle structure accounts for 60% of the pull-out strength, the vertebral body accounts for 15–20%, and precise fixation of a screw to the cortical bone of the anterior vertebral body accounts for the remaining 20–25%. Hirano et al. [15] also measured the pull-out strength of pedicle screws through biomechanical testing, and the tests revealed that 82% of the fixation strength and 57% of the pull out strength are attributable to vertebral pedicle structures. These studies thus established that the pedicle is the cornerstone for stable pedicle screw fixation.

Our results show that the pull-out strength of fixed screws decreases by approximately 25% when lateral perforation occurs, which differs slightly from the results of previous studies. This difference could be attributed to our slightly reformed study design, which aimed to mimic real-life surgical practice. In addition, in previous studies, only misplaced screws were considered, whereas in our study, the experiments focused on simulating the frequent surgical occurrence in which a surgeon manages to place the screw in the right tract even after perforating the lateral cortex. Our clinical experience suggests that lateral screw misplacement can be avoided with intraoperative diligence and that tactile feedback to the surgeon obtained by probing the drilled tract plays a vital role in the same. In the current study, although the screws appeared to have been well contained, lateral wall perforation compromised stability. The perforation led to loss of the integrity of the cylindrical structure of the vertebral pedicle, causing exposure of the screw thread, which in turn reduced the holding strength of the screw and weakened its fixation strength at length.

To achieve pedicular screw fixation for the lumbar spine, there are two key technical elements. First and foremost is the need to ensure an accurate entry point, and secondly, the principles of the appropriate transverse screw angle (TSA) must be followed. Thorough exposure and effective hemostasis are needed to find the right point. If the entry point is too lateral, the probe will perforate laterally at the very beginning. Then, the surgeon can realize the mistake with the pedicle feeler and shift the entry point medially accordingly. From our experience, we propose that the TSA should be 5–10° for L1–L3 and 10–15° for L4–L5, and the risk of breakage of the pedicular lateral wall will increase if the TSA is below the lower limit. However, ensuring optimal placement through a correct TSA to avoid pedicle perforation is not easy. Schizas [30] reviewed 130 studies published in the past 40 years, and in this meta-analysis, they found that without using navigation, only 86.5% of pedicle screws were accurately placed in the lumbar spine of cadaveric specimens, and this rate was only 87.3% in vivo. Tian [31] found that lumbar pedicle screw malposition is frequently accompanied by vertebral axial rotation, which is more common than anatomical variation and has a significant impact on TSA. Accordingly, the incidence of pedicle perforation will increase if surgeons do not pay enough attention to the change in TSA due to vertebral rotation [32,33]. Therefore, finding the rotation and selecting the appropriate TSA accordingly is the key to avoiding lateral perforation.

The study does have some limitations particularly in the terms of the small sample size and the inherent limitations associated with a cadaveric study. While the study shows that mechanical aberration occurs with lateral cortical perforation, it does not simulate the actual clinical scenarios in which these perforations may be repaired over time and may not affect the clinical outcomes. Additionally, as newer technologies such as computer-assisted surgery [34] and rapid prototyping [35,36] become universally available, the incidence of these inadvertent perforation may no longer remain relevant. However, currently, such perforation remains a key intraoperative problem, and this study highlights the need to be diligent during the surgery in order to avoid these complications.

## Conclusion

The integrity of the lumbar vertebral pedicle strongly affects the fixation strength of pedicle screws. Perforation of the lateral wall decreases the pull out strength of screws by 23.4% compared to the pull-out strength in the control group in which perforation did not occur.
